# Profound Effect of Profiling Platform and Normalization Strategy on Detection of Differentially Expressed MicroRNAs – A Comparative Study

**DOI:** 10.1371/journal.pone.0038946

**Published:** 2012-06-18

**Authors:** Swanhild U. Meyer, Sebastian Kaiser, Carola Wagner, Christian Thirion, Michael W. Pfaffl

**Affiliations:** 1 Physiology Weihenstephan, ZIEL Research Center for Nutrition and Food Sciences Technische Universität München, Freising, Germany; 2 Friedrich-Baur-Institute, Department of Neurology, Ludwig-Maximilians-Universität München, München, Germany; 3 Department of Statistics, Ludwig-Maximilians-Universität München, München, Germany; 4 IMGM Laboratories GmbH, Martinsried, Germany; 5 SIRION Biotech, Martinsried, Germany; The John Curtin School of Medical Research, Australia

## Abstract

**Background:**

Adequate normalization minimizes the effects of systematic technical variations and is a prerequisite for getting meaningful biological changes. However, there is inconsistency about miRNA normalization performances and recommendations. Thus, we investigated the impact of seven different normalization methods (reference gene index, global geometric mean, quantile, invariant selection, loess, loessM, and generalized procrustes analysis) on intra- and inter-platform performance of two distinct and commonly used miRNA profiling platforms.

**Methodology/Principal Findings:**

We included data from miRNA profiling analyses derived from a hybridization-based platform (Agilent Technologies) and an RT-qPCR platform (Applied Biosystems). Furthermore, we validated a subset of miRNAs by individual RT-qPCR assays. Our analyses incorporated data from the effect of differentiation and tumor necrosis factor alpha treatment on primary human skeletal muscle cells and a murine skeletal muscle cell line. Distinct normalization methods differed in their impact on (i) standard deviations, (ii) the area under the receiver operating characteristic (ROC) curve, (iii) the similarity of differential expression. Loess, loessM, and quantile analysis were most effective in minimizing standard deviations on the Agilent and TLDA platform. Moreover, loess, loessM, invariant selection and generalized procrustes analysis increased the area under the ROC curve, a measure for the statistical performance of a test. The Jaccard index revealed that inter-platform concordance of differential expression tended to be increased by loess, loessM, quantile, and GPA normalization of AGL and TLDA data as well as RGI normalization of TLDA data.

**Conclusions/Significance:**

We recommend the application of loess, or loessM, and GPA normalization for miRNA Agilent arrays and qPCR cards as these normalization approaches showed to (i) effectively reduce standard deviations, (ii) increase sensitivity and accuracy of differential miRNA expression detection as well as (iii) increase inter-platform concordance. Results showed the successful adoption of loessM and generalized procrustes analysis to one-color miRNA profiling experiments.

## Introduction

MicroRNA (miRNA) expression profiling has become a standard bioanalytical technique and provides a first important step in characterizing the role of miRNAs, a class of small (21–24 nucleotides) noncoding RNAs which regulates gene expression at the posttranscriptional level (reviewed in [1]). Many studies which comprise global miRNA detection and quantification rely on oligo microarray-based methods (microarray technology) [2], [3]. Microarray methods have the advantage of being relatively low cost (reviewed in [4]), relatively quick from RNA labeling to data generation and simple to use [5] compared to e.g. ultra high-throughput sequencing technologies. MicroRNA microarray results are similar to mRNA expression profiling results most commonly validated by RT-qPCR which is referred to as ‘gold-standard’ for holistic relative miRNA quantification [6]. PCR-based platforms for miRNA expression profiling, which combine simultaneous analysis of a large number of targets in a single experiment and advantages of qPCR, are of high interest and very effective.

Unlike for mRNA expression microarrays, comprehensive quality control and standardization studies [7] are rather limited for microRNA microarrays. Furthermore, the usual assumptions employed for mRNA expression array normalization may not hold true for miRNA arrays as summarized by Sarkar et al. [8]. Studies addressing intra-platform repeatability and inter-platform comparability of different miRNA microarray platforms [9] or microarray versus RT-qPCR profiling platforms [3], [10] are rare. However, selection of normalization methods for miRNA microarrays can have effects on resulting data outcome [8], [11]–[13] and physiological interpretation as adequate normalization methods can minimize the effects of systematic experimental bias and technical variations (reviewed in [14]). Optimal normalization of miRNA data may even be more critical than that of other RNA functional classes since relatively small changes in miRNA expression may be biologically and clinically significant [15], [16]. Moreover, the recently defined MIQE guidelines for quality control and standardization of RT-qPCR experiments [17] imply the use of the optimal normalization method. There is no clear consensus on the relative performance of normalization methods for miRNA profiling data as results and recommendations from previous studies have been inconsistent [18], [19]. Further comparative studies providing guidance or suggestions of adequate normalization to the community are needed to facilitate the application of adequate miRNA normalization methods and provide an estimate for cross-platform comparisons.

Thus, the objective of this study was to investigate the impact of normalization methods on intra- and inter-platform performance of distinct miRNA profiling approaches. We hypothesized that selection of an appropriate normalization method could minimize standard deviations, increase sensitivity, and cross-platform similarity of miRNA expression and thus increase intra- and inter-platform comparability and validity.

**Figure 1:**
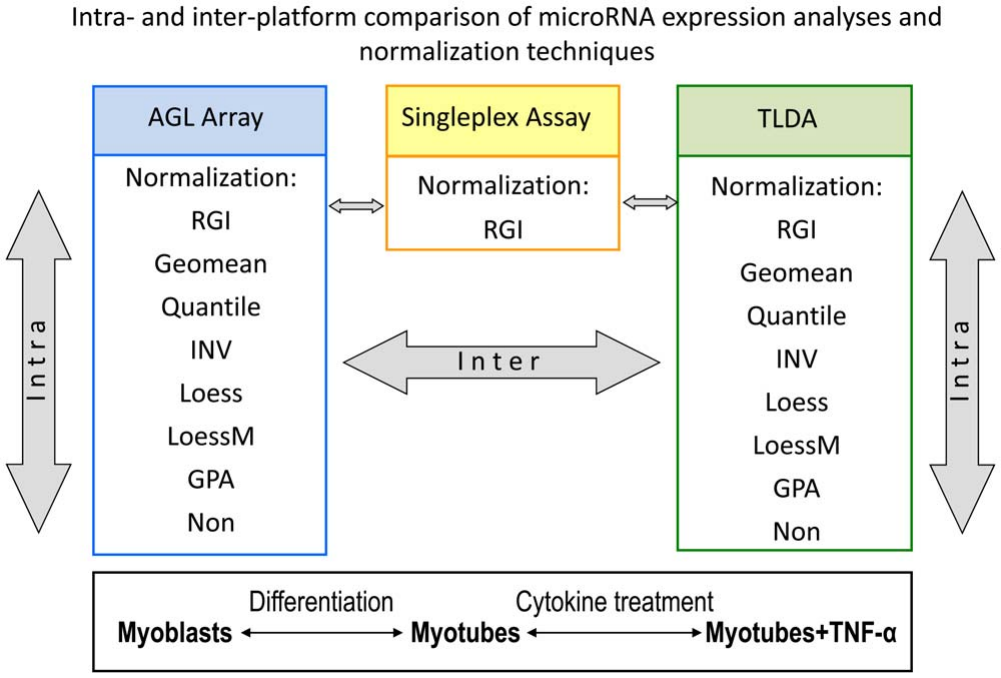
Platforms and normalization methods applied. Overview of intra- and inter-platform comparisons using miRNA microarrays from Agilent Technologies (AGL Array) and RT-qPCR arrays from Applied Biosystems (TLDA) for human and mouse miRNAs as well as singleplex TaqMan miRNA assays. Different normalization methods were applied to the platforms. Three distinct biological stages of mouse and primary human skeletal cells were analyzed.

This study evaluated the impact of normalization strategies on a hybridization-based platform from Agilent Technologies (Santa Clara, USA) (AGL array) and a multiplex/megaplex RT-qPCR platform from Applied Biosystems (Foster City, USA) (TLDA) relative to singleplex RT-qPCR (Figure 1). We utilized normalization methods commonly used in one-color miRNA microarray or RT-qPCR profiling studies (reference gene index (RGI), global geometric mean (geomean), quantile, invariant selection (INV), loess [12], [20], [21], respectively, and adapted the LoessM normalization [11] and the assumption-free general procrustes analysis (GPA) [22] to one-color miRNA profiling platforms. The biological effect of cell differentiation and cytokine treatment on miRNA expression implemented the basis for inter- and intra-platform assessments. Patient derived primary human skeletal myoblasts and the murine skeletal muscle cell line PMI28 were cultured as undifferentiated myoblasts, differentiated myotubes and myotubes which had been treated with TNF-α *in vitro*. Thus, we could provide a comparative study of the impact of normalization methods over three different biological backgrounds, two species, and two profiling platforms (Figure 1).

## Results and Discussion

### Intra-platform Identification and Concordance of Differential miRNA Expression Depended on the Normalization Method

Both, oligonucleotide hybridization-based and RT-qPCR-based techniques are widely used for miRNA expression profiling. Considerable effects of normalization on the detection of differentially expressed genes have been reported for one- and dual-channel miRNA microarrays [9], [11]. Therefore, one objective of this study was to compare and evaluate the impact of RGI, geomean, quantile, INV, loess, loessM, and GPA normalization strategies on AGL array and TLDA data. We investigated the reduction of bias, the quality (diagnostic performance of the test) and quantity in identifying differentially expressed miRNAs as well as the dissimilarity of datasets after normalization.

#### Qualitative and quantitative effects of distinct normalization methods on the identification of differential miRNA expression within platforms

**Figure 2:**
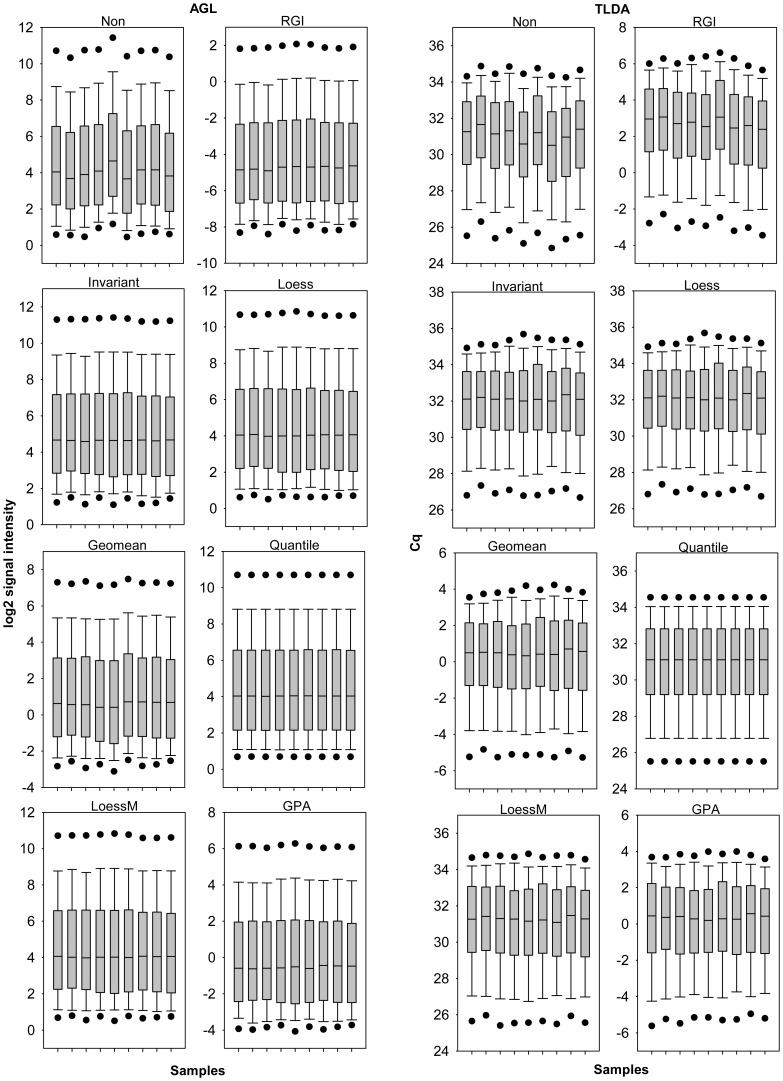
Signal distribution of human microarray and qPCR profiling. Box-whisker plot with 5th and 95th percentiles (black dots) of log2-transformed human AGL array signals or Cq values of human TLDA platform were shown for nine samples each across different normalization techniques.

**Figure 3:**
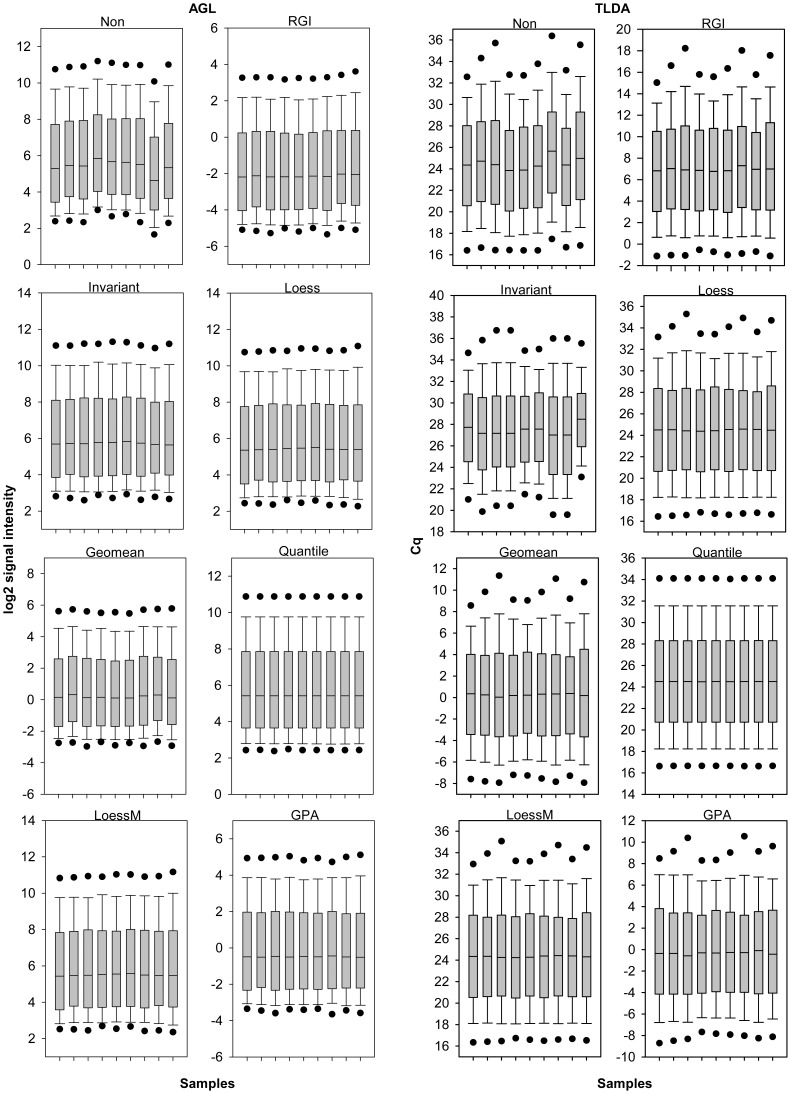
Signal distribution of mouse microarray and qPCR profiling. Box-whisker plot with 5th and 95th percentiles (black dots) of log2-transformed mouse AGL array signals or Cq values of mouse TLDA platform were shown for nine samples each across different normalization techniques.

We assume that a good normalization method should minimize the effects of systematic experimental and technical bias as well as reduce the variance between replicates. Signal distributions within Agilent arrays and TLDA cards were more similar after normalization compared to the non normalized datasets (Figure 2 and 3). The mean inter-replicate standard deviations of the three biological treatment groups and two different species were reduced by all normalization methods applied (Table 1 and 2). For the Agilent platform loess and loessM were most effective in reducing intra-group variation followed by INV, quantile, and GPA normalization. TLDA profiling revealed the least variation between replicates for loessM normalization followed by loess, quantile, and GPA normalization. Quantile normalization of TLDA data was reported to be more effective in reducing standard deviations than geomean normalization [23] which is in line with our data.

**Table 1:** Mean inter-replicate variance was minimized by applying normalization methods to the AGL array.

Normalization	mean	± sdev
RGI	0.183	0.064
Geom	0.204	0.073
**Quantile**	**0.176**	0.059
**INV**	**0.176**	0.059
**Loess**	**0.173**	0.061
**LoessM**	**0.173**	0.061
GPA	0.178	0.056
Non	0.320	0.163

**Agilent - Intra-platform.**

**Standard deviation.**

The average of intra-replicate standard deviations in human and mouse myoblasts, myotubes, and cytokine treated myotubes based on the platform-specific miRNA datasets were depicted. The mean intra-platform standard deviations depended on the normalization method.

**Table 2:** Mean inter-replicate variance was minimized by applying normalization methods to TLDA cards.

Normalization	mean	± sdev
RGI	0.662	0.119
Geom	0.651	0.126
**Quantile**	**0.642**	0.118
INV	0.696	0.220
**Loess**	**0.636**	0.115
**LoessM**	**0.635**	0.112
GPA	0.650	0.124
Non	0.738	0.166

**TLDA - Intra-platform.**

**Standard deviation** Legend information as specified for Table 1.

Moreover, we evaluated the impact of different normalization methods on sensitivity and specificity using the receiver operating characteristic (ROC) curve which is a plot of sensitivity (true positive rate) versus the formula 1 - specificity (or false positive rate). The area under the ROC curve (AUC) can be interpreted as a summary index of classification performance [24] between biological groups since it is a threshold independent global performance measure [25]. The effectiveness in distinguishing true differential expression due to myoblast differentiation or TNF-α treatment in human and mouse was best for loess, loessM, GPA, and INV normalization on both profiling platforms, AGL array and TLDA, as indicated by the mean AUC (Table 3 and 4). Based on the mean AUC, RGI normalization as well as geomean and quantile normalization turned out to be inferior in retaining treatment effects on the AGL array platform. Taken together, loess, loessM, GPA, and INV normalization robustly maximized sensitivity and specificity of classification in contrary to quantile normalization which was effective in the reduction of bias only. In line with our results Risso et al. [11] showed that loessM, GPA, and loessM combined with GPA outperformed quantile normalization in terms of sensitivity and specificity.

**Table 3:** Mean area under the ROC curve of AGL arrays.

Normalization	AUC mean	± sdev
**Loess**	**0.912**	0.079
**LoessM**	**0.912**	0.079
Quantile	0.899	0.074
INV	0.908	0.082
**GPA**	**0.911**	0.077
RGI	0.872	0.111
Geom	0.886	0.110
Non	0.905	0.113

**Agilent - Intra-platform.**

**ROC curves** The average of the AUCs of ROC analyses in human and mouse myoblast differentiation and cytokine treatment were illustrated based on the platform-specific miRNA sets. The mean AUC was influenced by normalization algorithms.

**Table 4:** Mean area under the ROC curve of TLDA cards.

Normalization	AUC mean	± sdev
**Loess**	**0.861**	0.154
**LoessM**	**0.861**	0.154
Quantile	0.856	0.155
**INV**	**0.889**	0.122
**GPA**	**0.884**	0.126
RGI	0.856	0.161
Geom	0.858	0.146
Non	0.841	0.170

**TLDA - Intra-platform.**

**ROC curves** Legend information as specified for Table 3.

Our study indicates, that normalization methods which turned out to increase the area under the ROC curve most effectively (loess, loessM, GPA, INV) resulted in an increase of significantly expressed miRs compared to no normalization for AGL array derived data (Table 5) and a reduction on TLDA derived data (Table 6), respectively. Latter illustrated that accumulation of systematic experimental or technical bias within replicates can either amplify or mask differential expression depending on the direction of regulation. Thus, applying normalization increase or decrease the dynamic (compare Figure S1) and significance of differential expression (Table 5 and 6). Interestingly, RGI normalization revealed the highest number of differentially expressed miRs on both platforms and a comparatively small area under the ROC curve. The trade-off between the true positive rate and specificity points to why superior normalization methods do not necessarily increase the amount of differentially expressed genes. At the same time the optimal normalization strategy for a platform’s dataset should yield a reasonable number of differentially expressed miRNAs since an overly aggressive normalization technique would cause an “averaging-out” effect [26].

**Table 5:** Effect of normalization methods on the number of differentially expressed miRNAs detected by AGL arrays.

Normalization	signifcant miRNAs	± sdev
RGI	83	38
Geom	72	35
Quantile	81	37
INV	81	37
Loess	81	37
LoessM	81	37
GPA	79	36
Non	54	31

**Agilent - Intra-platform.**

**Significant miRNAs** The mean number of differentially expressed miRNAs which were identified in distinctively normalized human and mouse myoblast differentiation and cytokine treated samples were depicted.

**Table 6:** Effect of normalization methods on the number of differentially expressed miRNAs detected by TLDA cards.

Normalization	signifcant miRNAs	± sdev
RGI	47	28
Geom	42	26
Quantile	37	30
INV	21	11
Loess	38	29
LoessM	38	29
GPA	31	20
Non	44	36

**TLDA - Intra-platform.**

**Significant miRNAs** Legend information as specified for Table 5.

The impact of normalization methods on the detection of differential expression was further evaluated by utilizing the Jaccard index [27] as similarity measure of differentially expressed gene lists. The non normalized datasets of the Agilent microarray and TLDA platform showed the tendency to reveal dissimilarity to the corresponding normalized datasets (Table 7 and 8, Figure S2 and S3) which is consistent with a general impact of normalization on data distribution, variance and detection of differential expression as discussed above. In the case of the AGL array, loess, loessM, GPA, and INV normalized datasets tended to show similarity in the detection of differentially expressed miRs (Table 7 and 8). The qPCR profiling platform revealed similarity among loess, loessM, GPA, and quantile normalized data. Thus, algorithms such as loess or loessM which are capable of removing intensity-dependent bias and the assumption free GPA algorithm robustly optimized datasets derived from Agilent microarrays and TLDA cards. Results from our study suggest that INV normalization performs better than quantile, RGI or geomean normalization on the Agilent microRNA platform. Pradervand et al. [12] suggested that normalization based on the set of invariants or quantile were more robust than e.g. scaling. Our study revealed that quantile normalization performed acceptable well for TLDA profiling data. However, we cannot confirm that quantile is one of the most robust normalization strategies as suggested by Rao et al. [26] and Zhao et al. [19] for miRNA microarrays.

**Table 7:** Impact of normalization strategies on the similarity of differential miRNA expression of AGL array data.

	Loess	LoessM	Quantile	INV	GPA	RGI	Geom	Non
**Loess**	1							
**LoessM**	**1**	1						
**Quantile**	0.681	0.681	1					
**INV**	**0.815**	**0.815**	0.652	1				
**GPA**	**0.787**	**0.787**	**0.725**	**0.742**	1			
**RGI**	0.652	0.652	0.638	0.669	0.723	1		
**Geom**	0.576	0.576	0.555	0.547	0.635	0.631	1	
**Non**	0.397	0.397	0.340	0.408	0.355	0.303	0.303	1

**Agilent - Intra-platform Jaccard index** The mean Jaccard indices of significantly regulated miRNA overlap across normalized datasets were depicted for myoblast differentiation and cytokine treated samples analyzed of human and mouse. The Jaccard index ranges between zero and one per definition. The closer the Jaccard index is to one the higher the relative similarity and reproducibility of differential expression across platforms.

Finally, geomean or RGI normalization did not perform acceptable well neither on the microarray nor on the qPCR platform. Our data exemplifies that less sophisticated methods like geomean or RGI normalization which can only correct for ‘global multiplicative effects’ might not be sufficient for miRNA profiling data.

**Table 8:** Impact of normalization strategies on the similarity of differential miRNA expression of TLDA card data.

	Loess	LoessM	Quantile	INV	GPA	RGI	Geom	Non
**Loess**	1							
**LoessM**	**1**	1						
**Quantile**	**0.723**	**0.723**	1					
**INV**	0.598	0.598	0.513	1				
**GPA**	**0.741**	**0.741**	**0.677**	0.597	1			
**RGI**	0.557	0.557	0.524	0.408	0.526	1		
**Geom**	**0.680**	**0.680**	0.606	0.495	**0.691**	0.596	1	
**Non**	0.560	0.560	0.476	0.468	0.520	0.425	0.421	1

**TLDA - Intra-platform Jaccard index** Legend information as stated for Table 7.

#### Assessment of assumptions underlying distinct normalization methods

The adequacy of normalization approaches might depend on whether the datasets meet the assumptions which underlie the respective algorithms. Normalization methods such as quantile and loess are based on two assumptions, (i) only a small portion of miRNAs is differentially expressed, and (ii) differentially expressed spots are homogeneously distributed with respect to both, over- and under-expressed miRNAs [11]. However, these assumptions could fail for miRNA profiling data [11], [13]. Since the number of expressed miRNAs in a given sample tends to be much smaller than that observed when profiling mRNA expression the proportion of those that are differentially expressed (among those expressed at all) is much larger compared to mRNA [8]. We verified whether the above assumptions hold true for our datasets. In this study 44.4% (162 miRNAs) human and 70.1% (410) rodent miRNAs of the theoretically detectable miRNAs within the TLDA platform passed the quality control criteria and were considered as successfully detected. The AGL array platform detected 37.8% (302 miRNAs) human and 40.5% (282) murine miRNAs of the theoretically detectable miRNAs. On average, 26.3% of the expressed miRNAs were differentially regulated within the AGL array data and 13.0% within the TLDA card data during myoblast differentiation and cytokine treatment in human and mouse. Thus, the proportion of differentially regulated miRNAs is in the range revealed by other miRNA profiling studies and less than 50% as summarized in Rao et al. [26]. However, even if one would expect a significant fraction to be differentially expressed INV normalization is particularly appropriate since INV-based regression assumes that there is a subpopulation of expressed genes that does not change [12]. Furthermore, the assumption of symmetry of differentially expressed miRNAs was investigated. Symmetry of up- and down-regulations for the human and murine samples was overall balanced across normalization methods and the non-normalized dataset as reflected by a mean of log_2_ regulations close to zero namely 0.07±0.22 (differentiation) and −0.18±0.26 (cytokine treatment) on AGL array platform and overall symmetry on the TLDA platform with a mean Cq regulation of 0.29±0.3 upon differentiation and 0.12±0.51 due to the effect of cytokine treatment. Moreover, normalization strategies such as loessM do not depend on the assumptions that there exists only a small proportion of differentially expressed miRNAs and the distribution of differential miRNA expression is symmetrical between over and under expressed [11]. Finally, GPA is an assumption free approach [22]. Quantile normalization assumes that the overall distribution of signal intensity does not change which is the case for AGL array data as well as TLDA data in this study (Figure 2 and 3). Generally speaking, all normalization methods applied within this study were applicable for the datasets presented here and should be compatible for the vast majority of studies using one-color hybridization or RT-qPCR based miRNA profiling platforms.

### Platform-specific Selection of Normalization Strategies can Maximize Inter-Platform Concordance of Differential miRNA Expression

The confirmation of differential expression by independent and rather different profiling approaches is of particular interest in miRNA research. Since comparatively small changes in miRNA expression might be of physiological relevance the verifiability of miRNA expression across platforms is a useful approach to get a first estimate of the biological importance. To evaluate inter-platform concordance of relative miRNA expression we investigated subsets of 127 miRNAs from human and 201 miRNAs from mouse samples which contained all miRNAs successfully detected by both, AGL array as well as TLDA platform.

#### Intra-platform performance of normalization methods was confirmed for the platform overlapping miRNA subset

The impact of different normalization methods on the quality and quantity of differential expression within the miRNA overlapping subsets was evaluated. Thereby we wanted to exclude the effect of a putative subset specific performance of normalization methods. We could confirm similar tendencies of normalization performance measures in the platform overlapping number of miRNAs (common inter-platform subset) compared to the observations for the platform specific miRNA subset described above. Standard deviations of the six biological groups (three human and three mouse groups) were reduced by all normalization methods applied (Table S1 and S2) compared to the non normalized datasets. The reduction of standard deviations on the Agilent platform was most evident for loess and loessM. Moreover, INV, quantile, RGI, and GPA normalization were effective in alleviating standard deviations. Variation within the TLDA platform was lessened most by loessM normalization followed by loess, quantile, and GPA normalization. The overall sensitivity and specificity in detection of differential gene expression was best for loess, loessM, GPA, and INV normalized Agilent microarray and TLDA data. Loess, loessM, GPA, and INV normalization shifted the trade-off between true positive rate and false positive rate towards higher mean AUCs (Table 9 and 10). The numbers of significantly regulated miRNAs were similarly influenced by the normalization approaches (Table S3 and S4) as described for the platform-specific datasets. Since the stability of lowess smoothers is known to be dependent on the number of data points to which they are applied [19] it is worth noting that loess and loessM seem to robustly improve data quality on different sizes of datasets (as shown for the platform-specific as well common miRNA subset). In concert with the platform-specific data, results from the platform-shared miRNA sets underscore the importance of adequate evaluation and selection of the normalization method which had distinct impact on the quantity and accuracy of differential miRNA expression.

**Table 9:** Mean area under the ROC curve of the inter-platform miRNA subsets for AGL arrays.

Normalization	AUC mean	± sdev
**Loess**	**0.923**	0.080
**LoessM**	**0.923**	0.080
Quantile	0.903	0.069
**INV**	**0.919**	0.086
**GPA**	**0.915**	0.084
RGI	0.875	0.148
Geom	0.899	0.098
Non	0.902	0.121

**Agilent - Inter-platform.**

**ROC curves** The average of the AUCs of ROC analyses in human and mouse myoblast differentiation and cytokine treatment were shown for the miRNA subset shared by both platforms. The mean AUC was influenced by normalization algorithms similar to the platform-specific dataset.

**Table 10:** Mean area under the ROC curve of the inter-platform miRNA subsets for TLDA cards.

Normalization	AUC mean	± sdev
**Loess**	**0.921**	0.094
**LoessM**	**0.921**	0.094
Quantile	0.903	0.102
**INV**	**0.920**	0.116
**GPA**	**0.923**	0.092
RGI	0.912	0.094
Geom	0.910	0.095
Non	0.909	0.138

**TLDA - Inter-platform.**

**ROC curves** Legend information as stated for Table 9.

#### Influence of distinct normalization methods on inter-platform concordance of differential miRNA expression

Validation of miRNA microarray data by an independent method such as qPCR has been widely used and accepted as gold standard. However, platform-specific bias and performance characteristics might impact consistency across platforms. We propose that adequate platform-specific normalization methods could maximize inter-platform concordance of differential miRNA expression. Inter-platform similarity of miRNA expression regulation was evaluated by calculating the Jaccard indices between platforms and corresponding normalization approaches (Table 11, Figure S4) for the common miRNA subset. The comparison of Jaccard indices for myoblast differentiation and cytokine treatment in human and mouse showed a tendency of loess, loessM, quantile, and GPA normalization of AGL data to increase similarity across platforms. For the TLDA derived data the results of similarity analysis indicate that loess, loessM, geomean and RGI increased consistency and reproducibility of differentially expressed miRNAs across platforms. The following combinations substantially increased inter-platform concordance of differential expression as listed in descending order: AGL-non with TDLA-non, AGL-GPA with TLDA-geom, AGL-loess/loessM with TLDA-loess/loessM, AGL-quant with TLDA-RGI (Table 11). Based on the Jarrad index the least inter-platform concordance was achieved among RGI normalized Agilent data and INV normalized TLDA data. Comparatively high Jaccard indices between the non normalized datasets of Agilent microarray and non normalized TLDA cards might be explained by the similar number of differentially expressed miRNAs (Table S3 and S4). As the Jaccard index gives the intersection of differentially expressed miRNA lists relative to the union of the miRNA lists the divisor in this calculation is comparatively small for the non normalized datasets giving a high similarity measure. However, the absolute number of miRNAs consistent between the non normalized data is smaller on average compared to the list overlap of normalized datasets (Figure S5). Hence, adequate selection of normalization methods such as loess or loessM could increase the similarity of inter-platform validated miRNAs. Geomean normalization of TLDA data showed the tendency to increase inter-platform concordance, but on the basis of our intra-platform data we can favour geomean normalization for TLDA profiling data only when cross-platform validation is available to avoid false positives. Interestingly, the subset of differentially expressed miRNAs which were reproducibly identified across normalized platforms included miRNAs that had been functionally validated to play a role in skeletal muscle [28], [29]. Taken together, our data underscores that adequate normalization can increase inter-platform comparability and validity. Thus, normalization might be an important adjustable factor in the verifiability and consistency of miRNA expression across platforms.

**Table 11:** Inter-platform concordance of differential expression and its dependency on normalization methods.

TLDA	AGL							
	Loess	LoessM	Quantile	INV	GPA	RGI	Geom	Non
**Loess**	**0.292**	**0.292**	**0.280**	0.271	**0.282**	0.230	0.268	0.272
**LoessM**	**0.292**	**0.292**	**0.280**	0.271	**0.282**	0.230	0.268	0.272
**Quantile**	0.270	0.270	0.266	0.259	**0.280**	0.229	0.253	0.269
**INV**	0.218	0.218	0.212	0.198	0.213	0.174	0.181	0.267
**GPA**	0.260	0.260	0.267	0.244	0.275	0.210	0.248	0.246
**RGI**	0.276	0.276	**0.288**	0.257	**0.282**	0.227	0.240	0.245
**Geom**	**0.285**	**0.285**	0.279	0.259	**0.296**	0.237	0.271	0.242
**Non**	0.242	0.242	0.224	0.229	0.235	0.176	0.223	**0.331**

**Agilent and TLDA - Inter-platform Jaccard index** The average Jaccard indices of significantly regulated miRNA overlap across human and mouse AGL array and TLDA was depicted for the miRNA subset shared by both platforms. The closer the Jaccard index is to one the higher the relative similarity and reproducibility of differential expression across platforms.

### Adequate Normalization of Profiling Data Yields Good Verifiability by Singleplex Assays

#### Individual RT-qPCR assay analysis validated inter-platform concordance of differential expression

**Figure 4:**
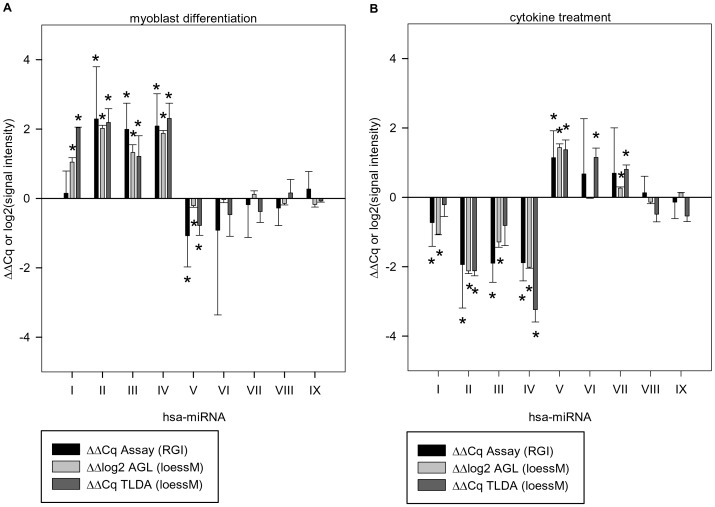
Differential expression detected by three different miRNA analysis approaches. Concordance and validation of ΔΔCq or ΔΔlog2 values, respectively, of three different human miRNA analysis methods: Singleplex RT-qPCR assay (RGI), AGL array (loessM), and TLDA (loessM). The effect of myoblast differentiation (A) and cytokine treatment (B) was investigated. Significant miRNA regulations were indicated by asterisks. Nine human miRNAs were represented by Latin numbers I–IX (see materials and methods for more detailed information).

We propose that adequate normalization of miRNA profiling data yields good verifiability by individual qPCR assays. Hence, we selected differentially expressed miRNAs (p<0.05) of the loessM-normalized common subset of human miRNAs on AGL array and TLDA and evaluated expression by individual assays for myoblast differentiation (Figure 4 A) and cytokine treatment effect (Figure 4 B). Inter-platform concordance of significantly regulated miRNAs was validated in the majority of cases (Figure 4 A miR-II to -V and 4 B miR-II, -IV, -V). Furthermore, miRNAs which were not significantly regulated on either of the platforms could be confirmed by individual assays as well (Figure 4 A miR-VI to -IX, 4 B miR-VIII, -IX). Correlation coefficients of individual assays and loessM normalized AGL array as well as TLDA were considerable high (Spearman’s R = 0.875, p<0.01) validating inter-platform concordance of differential expression.

### Platform-specific Characteristics of Datasets

#### Inter-platform differences of variability and dynamic of differential expression

The total number of differentially expressed miRNAs was larger for AGL arrays than for TLDA even if the same miRNA subsets were observed (Table S3 and S4). In general one would rather expect the RT-qPCR system to reveal a higher number of differential expressions due to high sensitivity of the system and template amplification of qPCR. However, the higher overall miRNA expression standard deviation of 0.635±0.112/0.413±0.070 (loessM normalized platform specific/common dataset) compared to AGL array with an average standard deviation of 0.173±0.061/0.137±0.077 (loessM normalized platform specific/common dataset) indicated that qPCR was associated with the amplification of bias as well. Moreover, the comparison of fold-changes indicated a compression of fold-change dynamic for the AGL array (Figure S1) compared to TLDA which is in line with results from Pradervand et al. (2009). Compression of differential gene expression across platforms was indicated by linear regression with a slope of 0.302/0.632 (human/mouse) for myoblast differentiation and 0.352/0.233 (human/mouse) for TNF-α treatment (Figure S6). However, comparison of t-values illustrated a compression of t-values for the TLDA platform compared to AGL array with a slope of −0.181/−0.375 (human/mouse) for myoblast treatment and −0.121/−0.414 (human/mouse) for TNF-α treatment (Figure S7). Hence, we might conclude that in our study the AGL array identified more differentially expressed genes due to less variance. Generally speaking, inter-platform similarity was rather low which is in line with a study by Chen et al. [3] reporting considerable variability between miRNA microarray and TLDA data indicated by low correlation between the two methods.

### Summary and Conclusions

This is the first comparative study evaluating the impact of RGI, geomean, INV, quantile, loess, loessM, and GPA normalization methods on intra-platform performance as well as inter-platform comparability of two commonly used platforms, a one-color hybridization-based Agilent microarray versus an RT-qPCR miRNA profiling platform from Applied Biosystems. We used mouse and human samples and validated profiling results by individual miRNA RT-qPCR assays. In summary, normalization reduced inter-replicate standard deviations and affected differential miRNA expression detection. Normalization methods like loess, loessM, GPA, and INV which increased sensitivity of classification did not maximize the number of differentially expressed miRNAs. Furthermore, the intra-platform performance of normalization methods was confirmed for the platform overlapping miRNA subset. In general, selection of the profiling platform affected the variability and dynamic of differential miRNA expression. However, the platform-specific selection of normalization strategies could maximize consistency and reproducibility of differential miRNA expression detection across profiling platforms and yielded good verifiability by singleplex qPCR assays. To put it in a nutshell, the choice of the normalization strategy had a qualitative and quantitative impact on the identification of differential miRNA expression and could contribute to the adjustment of platform-specific performance differences.

In conclusion, we recommend the application of loess, or loessM, and GPA normalization for miRNA Agilent hybridization arrays and qPCR TLDA cards. Loess, loessM, and GPA normalizations showed to (i) effectively reduce standard deviations, (ii) increase sensitivity and accuracy of differential miRNA expression detection as well as (iii) increase inter-platform concordance. This study showed the successful adoption of loessM and GPA to one-color miRNA profiling experiments. Our results provide an additional piece of evidence that the choice of the normalization algorithm and profiling platform has a profound effect on determining differential miRNA expression and we encourage researchers to evaluate the sensitivity of their data to different assumptions and algorithms.

## Materials and Methods

### Cell Culture

Primary human skeletal muscle cells (hSkMCs) were obtained from the “Muscle Tissue Culture Collection” at the Friedrich-Baur-Institute (LMU, Munich, Germany), and were propagated in skeletal muscle cell growth medium low serum (PromoCell) supplemented with 10% fetal calf serum (FCS) (PAA Laboratories), and 2 mM L-glutamine (PAA Laboratories). The murine skeletal myoblast cell line PMI28 [30] was cultured in Ham’s F10 (PAA Laboratories), supplemented with 20% FCS (Sigma-Aldrich), 2 mM L-glutamine (PAA Laboratories), and 1% Penicillin/Streptomycin (PAA Laboratories). Myoblasts were propagated at 37°C in humidified air (80% relative humidity) and 5% CO_2_. Human and murine myoblasts were cultured on laminin-1 coated dishes for an additional 24 h before switching a fraction of dishes to differentiation medium (DMEM medium containing 2% horse serum (Gibco), 2 mM L-glutamine (PAA Laboratories), and 0.1% gentamicin (Gibco) (human myoblasts) or 1% Penicillin/Streptomycin (murine myoblasts)) with 2×10^3^ U/ml human recombinant TNF-α (Roche Applied Science) or 2×10^3^ U/ml murine recombinant TNF-α (Roche Applied Science) or carrier, respectively. All media were replenished twice a day. hSkMCs and pmi28 cells were harvested 24 h after the induction of fusion by serum withdrawal.

### Total RNA Preparation

Human cell pellets were lysed and homogenized with Qiazol (Qiagen) and total RNA was extracted using the RNeasy Mini Kit (Qiagen) according to the manufacturer’s instructions. Murine cells were lyzed in Trizol (Invitrogen) and total RNA was prepared according to the manufacturer’s instructions. Total RNA concentrations were determined photometrically using the NanoDrop 1000 ND-1000 (Peqlab). RNA quality was characterized using the 2100 Bioanalyzer (Agilent Technologies) [24]. Samples yielded high RNA quality (RIN values between 8 and 10) and were further processed for profiling or individual qPCR analyses.

### MiRNA Microarray Analysis

MicroRNA expression profiling of myoblasts, myotubes and cytokine treated myotubes with three cell culture replicates per groups was performed by using an oligonucleotide hybridization-based platform from Agilent Technologies. Human samples were analyzed with the Human MicroRNA Microarray V2 (AGL array) containing probes for 723 human and 76 human viral miRNAs from Sanger miRBase 10.1. Murine samples were profiled with Mouse miRNA Microarray Release 15.0 containing probes for 696 miRNAs from Sanger miRBase release 15.0. We used 100 ng total RNA per sample and microarray. Labeling and hybridization was performed according to the manufacturer’s instructions. Resultant data from AGL arrays were extracted from image files and log_2_-transformed utilizing the Feature Extraction Software (Agilent Technologies). For further analysis only those miRNAs which showed a signal greater than zero in at least two of the three cell culture replicates within a group were retained thereby leaving 302 miRNAs for human and 282 miRNAs for murine samples. Different normalization approaches were applied (see section “Normalization” further below). All Agilent microarray data were MIAME compliant and were registered into ArrayExpress database [31], a publicly available repository consistent with the MIAME guidelines. Data are available with the following ArrayExpress accession numbers E-MTAB-299 (human dataset) and E-MTAB-1114 (mouse dataset).

### MiRNA RT-qPCR Profiling

The TaqMan Array Human MicroRNA Panel 1.0 (Applied Biosystems) (based on Sanger miRBase 9.2) facilitated the specific amplification and detection of 365 different mature human microRNAs by TaqMan-based quantitative real-time PCR in a 384-well or TaqMan Low Density Array format (TLDA). Outlining the experimental procedure, for each sample and plate eight separate multiplex reverse transcription (RT) reactions (Human Multiplex RT Set Pools 1–8) were performed with 50 ng total RNA each. Stem-loop structured RT primers allowed for the specific RT of mature miRNAs with single-base discrimination [32]. The resulting cDNA was loaded into the arrays and TaqMan real-time PCR was performed using the 7900 HT Fast Real-Time PCR System (Applied Biosystems) with cycling conditions according to the manufacturer’s protocol. 150 ng total RNA of murine pmi28 samples were reverse transcribed and preamplified using the MegaPlex Rodent Primer Pool Set (Life Technologies) according to the manufacture’s instructions. Preamplified samples were profiled with the TaqMan Rodent MicroRNA Arrays 2.0 (Life Technologies) including primers for 585 different mature miRNA. All samples analyzed by the Agilent platform were included in the TLDA analyses. TDLA profiling was conducted at IMGM Laboratories GmbH on Applied Biosystems 7900 HT Fast Real-Time System with cycling conditions according to the manufacturer’s instructions. Raw data was obtained using SDS 2.3 software (Applied Biosystems). All SDS files were analyzed utilizing the RQ Manager 1.2 software (Applied Biosystems). miRNAs meeting the detection criterion of showing Cq-values smaller 35 (human samples) or 32 (murine samples) in at least two of the corresponding triplicates of a group (as recommended by the vendor) were retained for further data processing. For the human and the murine samples each, a common subset of miRNAs passing pre-processing procedure on both, the AGL array and the TLDA platform, was identified based on nomenclature and/or sequence identity giving rise to a set of 127 human miRNAs and a common subset of 201 miRNAs for the mouse cells. Data was normalized as described in section “Normalization”.

### Validation of miRNA Profiling with RT-qPCR

Selected miRNAs were analyzed in myoblasts (n = 4), myotubes (n = 3) and myotubes treated with TNF-α (n = 3) using individual TaqMan MicroRNA Assays and reverse transcription reagents from Applied Biosystems according to the manufacturer’s instructions. Validation of microRNA profiling data by individual assays was performed in quadruplicate reverse transcription reactions and qPCR reactions for each cell culture replicate. MiRNAs selected for validation include three miRNAs with expression values corresponding to the median value of not normalized human TLDA data of myotubes. Two of these miRNAs with expression values consistent with the median were identified to be stably expressed by geNorm [33] analysis of TLDA data. Furthermore, a significantly regulated miRNA with expression levels below the median was included as well as five significantly regulated miRNAs (both, during differentiation and cytokine treatment or as response to cytokine treatment only) with expression values higher than the median. Most of the selected miRNAs corresponded to candidates in the upper half of expression values because significantly, and thus biologically interesting miRNAs, were identified primarily in the mentioned expression range.

### Normalization

We used seven different methods (RGI, geomean, quantile, INV, loess, loessM und GPA) to normalize the data. Since there is no gold standard for miRNA normalization, yet, we worked with all seven methods. The arithmetic mean of two stably expressed miRNAs as identified by GeNorm [33] or Normfinder [34] served as reference gene [35] index. Furthermore, the global geometric mean of all expressed miRNAs in one sample [20] which met the detection criteria was used for normalization. The standard normalizations quantile and loess are described by Bolstad et al. [21] and the invariant selection was introduced by Pradervand et al. [12]. For the loessM normalization we adapted the method of Risso et al. [11]. In order to avoid small values close to 0 the median of the respondent value in the loess estimation is added to the dataset. This modification relaxes the assumption of symmetry among up- and down-regulated genes [11]. Since this intra-array normalization method is normally used with two dimensional arrays (green and red signal) we adopted the method for our one dimensional arrays. A brief description and the corresponding R code can be found in the Technical Appendix and http://www.statistik.lmu.de/~kaiser/sup-material.html, respectively. LoessM normalization was applied since it is an assumption-free inter-array method. The same problem arose in the Generalized Procrustes Analysis (GPA). Since Xiong et al. [22] used the GPA for their two-dimensional red and green signal intensities we used the GPA on the three Groups (MB, MT, MT+TNF) of arrays instead. A detailed description and code is enclosed in the Technical Appendix and http://www.statistik.lmu.de/~kaiser/sup-material.html, respectively. We utilized GPA normalization since it is an assumption-free inter-array method.

The following normalizations were done in R [36] using the functions: normalize.quantiles (package preprocessCore, Bioconductor [37]), normalize.loess (package affy, Bioconductor [37]), normalize.loessM (own code, http://www.statistik.lmu.de/~kaiser/sup-material.html, Technical Appendix), invariant_selection.R (Supporting information of [12]), normalize.GPA (modified procGPA function from package shape, http://www.statistik.lmu.de/~kaiser/sup-material.html, Technical Appendix).

### Statistics

Significance of relative quantification [38] of miRNA expression levels was determined by applying significance analysis of microarrays (SAM) [39], an assumption free approach adopted to microarray. SAM identifies differentially expressed miRNAs by permutation.

## Supporting Information

Figure S1 **Fold-change distribution.** Distribution of fold-changes of human and mouse AGL microarray and TLDA platform data during myoblast differentiation and cytokine treatment were illustrated by box-whisker plots with 5^th^ and 95^th^ percentiles (black dots). Fold-change distribution of RGI, geomean, quantile, INV, loess, loessM, and GPA normalized and the non-normalized datasets were depicted.
(TIF)

Figure S2 **Human heatmap of relative similarity in detecting differential expression within distinctively normalized datasets.** Jaccard indices of significantly regulated miRNA overlap between distinctively normalized datasets were depicted for myoblast differentiation and cytokine treated samples analyzed on human AGL array or TLDA card. Colour coding of the heatmap was gradually from red indicating low similarity to white indicating a Jaccard index close to one.
(TIF)

Figure S3 **Mouse heatmap of relative similarity in detecting differential expression within distinctively normalized datasets.** Jaccard indices of significantly regulated miRNA overlap between distinctively normalized datasets were depicted for myoblast differentiation and cytokine treated samples analyzed on mouse AGL array or TLDA card. Colour coding of the heatmap was as stated in Figure S2.
(TIF)

Figure S4 **Heatmap of relative inter-platform similarity in detecting differential expression dependent on the normalization applied.** Jaccard indices of significantly regulated miRNA overlap across the two distinctively normalized platforms, AGL array and TLDA card, were depicted as heatmap for myoblast differentiation and cytokine treated samples. Colour coding of the heatmap was as stated in Figure S2.
(TIF)

Figure S5 **Inter-platform absolute concordance of differential expression upon cytokine treatment.** Inter-platform concordance of differential expression detected by human AGL array and TLDA across different normalization methods and no normalization were exemplarily shown for the effect of cytokine treatment. The overlapping number of miRNAs between datasets was depicted for all possible inter-platform combinations of distinctively normalized datasets.
(TIF)

Figure S6 **Fold-change compression by microarray profiling.** Inter-platform fold-change concordance of human and mouse TLDA and AGL platform of the miRNA subset common on both platforms was illustrated by scatter plot of mean values of fold-changes (log2 scale or Cq, respectively). A fold-change compression of AGL platform values relative to the TLDA platform was indicated by linear regression (black line) shown with 95% confidence band (blue line) and 95% prediction band (red line).
(TIF)

Figure S7 **Inter-platform concordance of t-values reveals compression of t-values by qPCR profiling.** Inter-platform concordance of t-values of human and mouse TLDA and AGL platform for the common miRNA subsets was illustrated by scatter plot. A compression of t-values of TLDA platform relative to the AGL platform was indicated by linear regression (black line) shown with 95% confidence band (blue line) and 95% prediction band (red line).
(TIF)

Table S1 **Normalization reduced mean inter-replicate variances within the platform-overlapping miRNA subsets of AGL arrays.** The average of intra-replicate standard deviations in human and mouse myoblasts, myotubes, and cytokine treated myotubes were illustrated based on the platform-overlapping miRNA datasets.
(XLSX)

Table S2 **Normalization reduced mean inter-replicate variances within the platform-overlapping miRNA subsets of TLDA cards.** Legend information as stated for Table S1.
(XLSX)

Table S3 **The number of differentially expressed miRNAs within the platform-overlapping miRNA subsets of AGL arrays.** The mean number of differentially expressed miRNAs which were identified in distinctively normalized human and mouse myoblast differentiation and cytokine treated samples within the platform-overlapping miRNA subsets were depicted.
(XLSX)

Table S4 **The number of differentially expressed miRNAs within the platform-overlapping miRNA subsets of TLDA cards.** Legend information as specified for Table S3.
(XLSX)
