# Supplementary figures and images for: Profound Effect of Profiling Platform and Normalization Strategy on Detection of Differentially Expressed MicroRNAs – A Comparative Study
Source: PLoS One. 2012 Jun 18;7(6):e38946. doi: 10.1371/journal.pone.0038946 (PMC3377731; doi:10.1371/journal.pone.0038946)

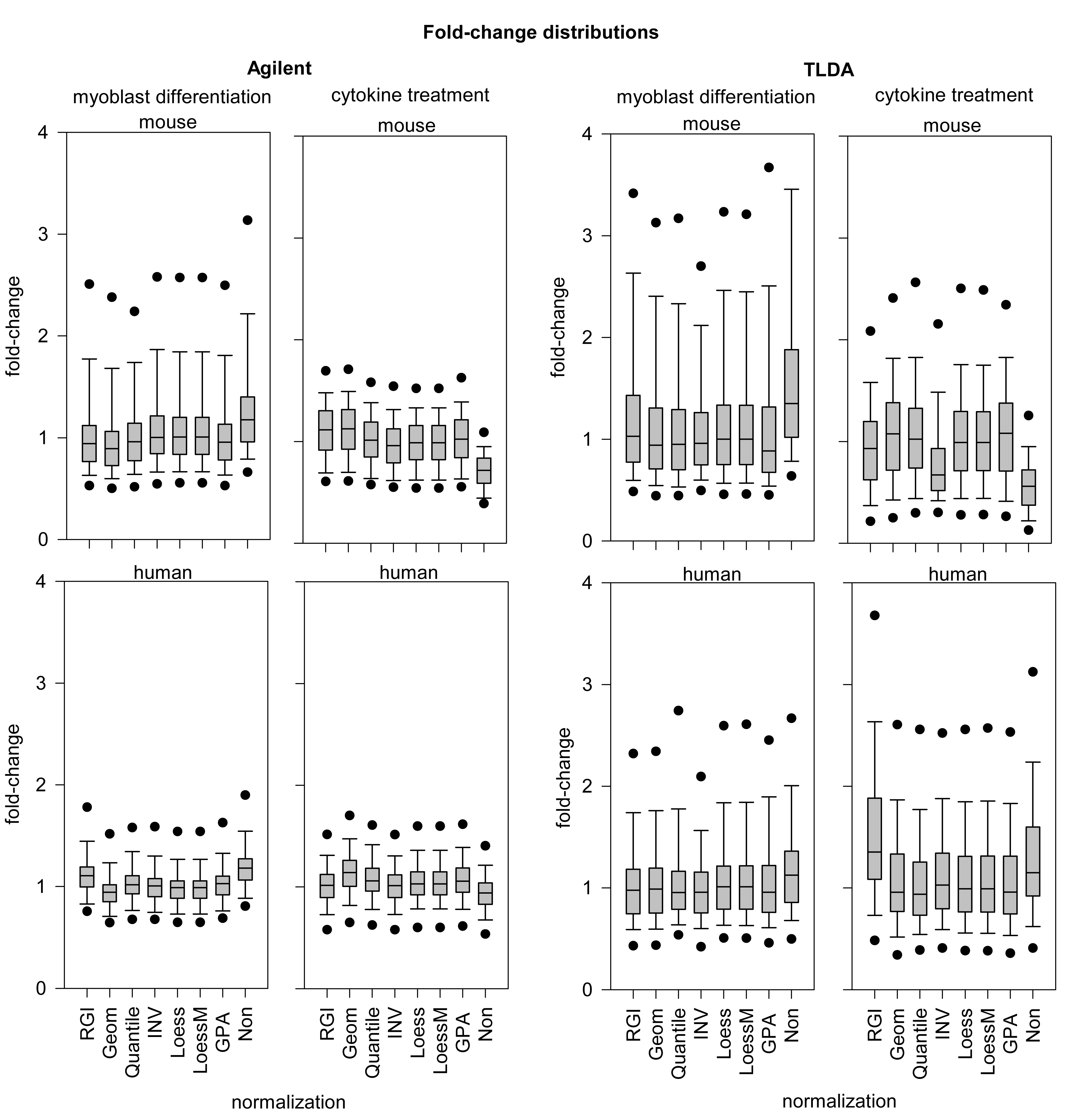

Supplement: Figure S1 — Fold-change distribution. Distribution of fold-changes of human and mouse AGL microarray and TLDA platform data during myoblast differentiation and cytokine treatment were illustrated by box-whisker plots with 5th and 95th percentiles (black dots). Fold-change distribution of RGI, geomean, quantile, INV, loess, loessM, and GPA normalized and the non-normalized datasets were depicted. (TIF) [file pone.0038946.s001.tif]

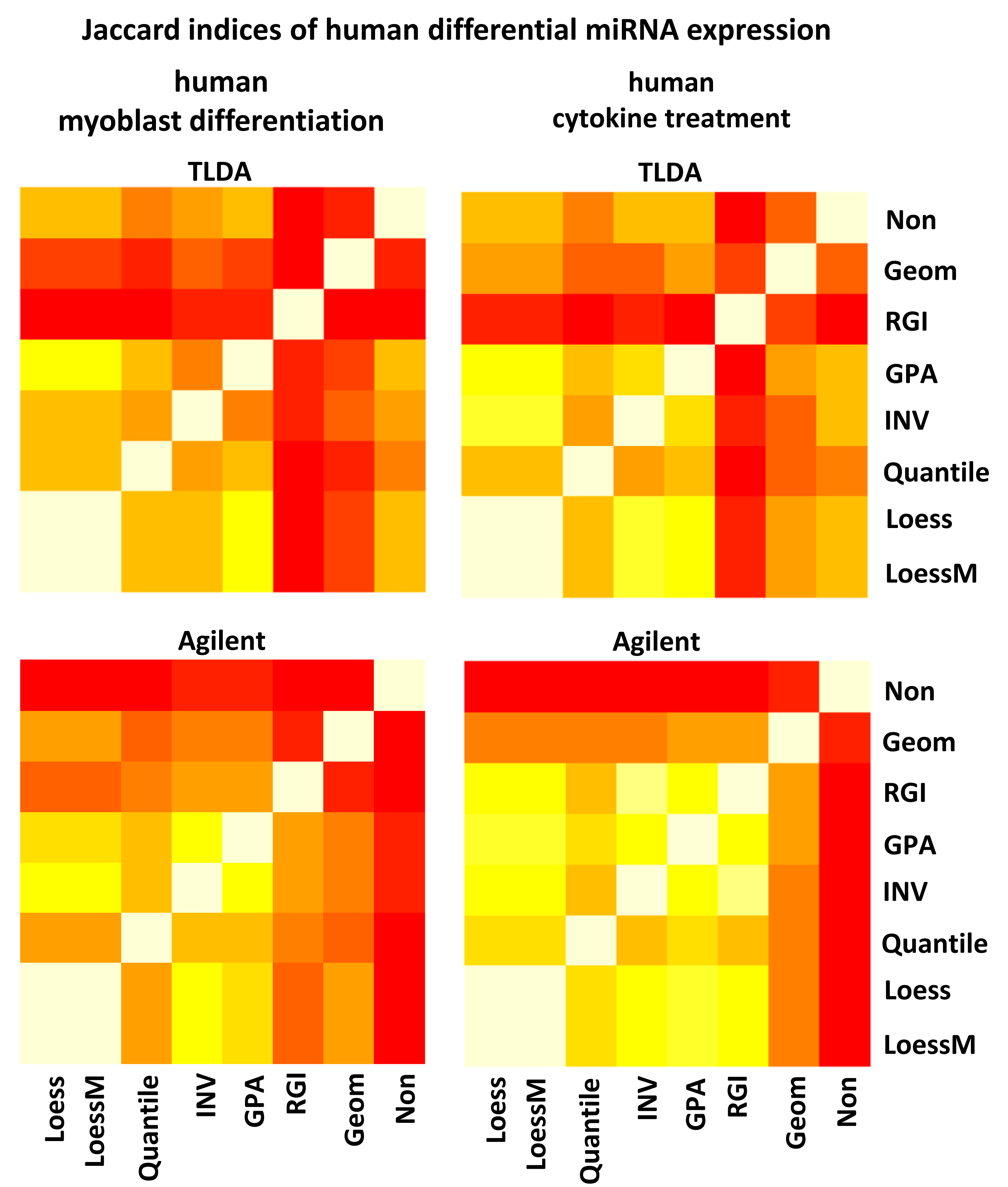

Supplement: Figure S2 — Human heatmap of relative similarity in detecting differential expression within distinctively normalized datasets. Jaccard indices of significantly regulated miRNA overlap between distinctively normalized datasets were depicted for myoblast differentiation and cytokine treated samples analyzed on human AGL array or TLDA card. Colour coding of the heatmap was gradually from red indicating low similarity to white indicating a Jaccard index close to one. (TIF) [file pone.0038946.s002.tif]

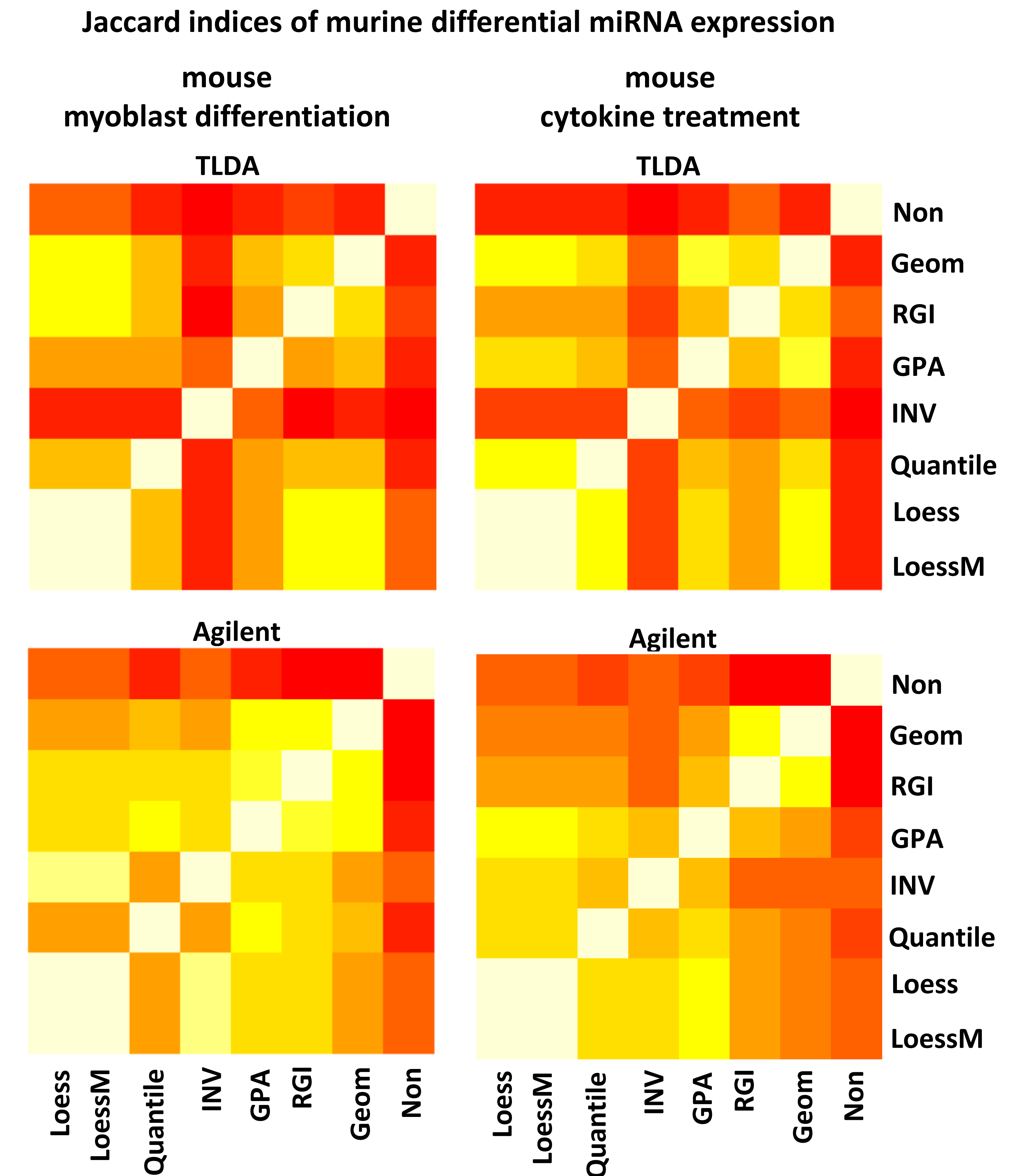

Supplement: Figure S3 — Mouse heatmap of relative similarity in detecting differential expression within distinctively normalized datasets. Jaccard indices of significantly regulated miRNA overlap between distinctively normalized datasets were depicted for myoblast differentiation and cytokine treated samples analyzed on mouse AGL array or TLDA card. Colour coding of the heatmap was as stated in Figure S2. (TIF) [file pone.0038946.s003.tif]

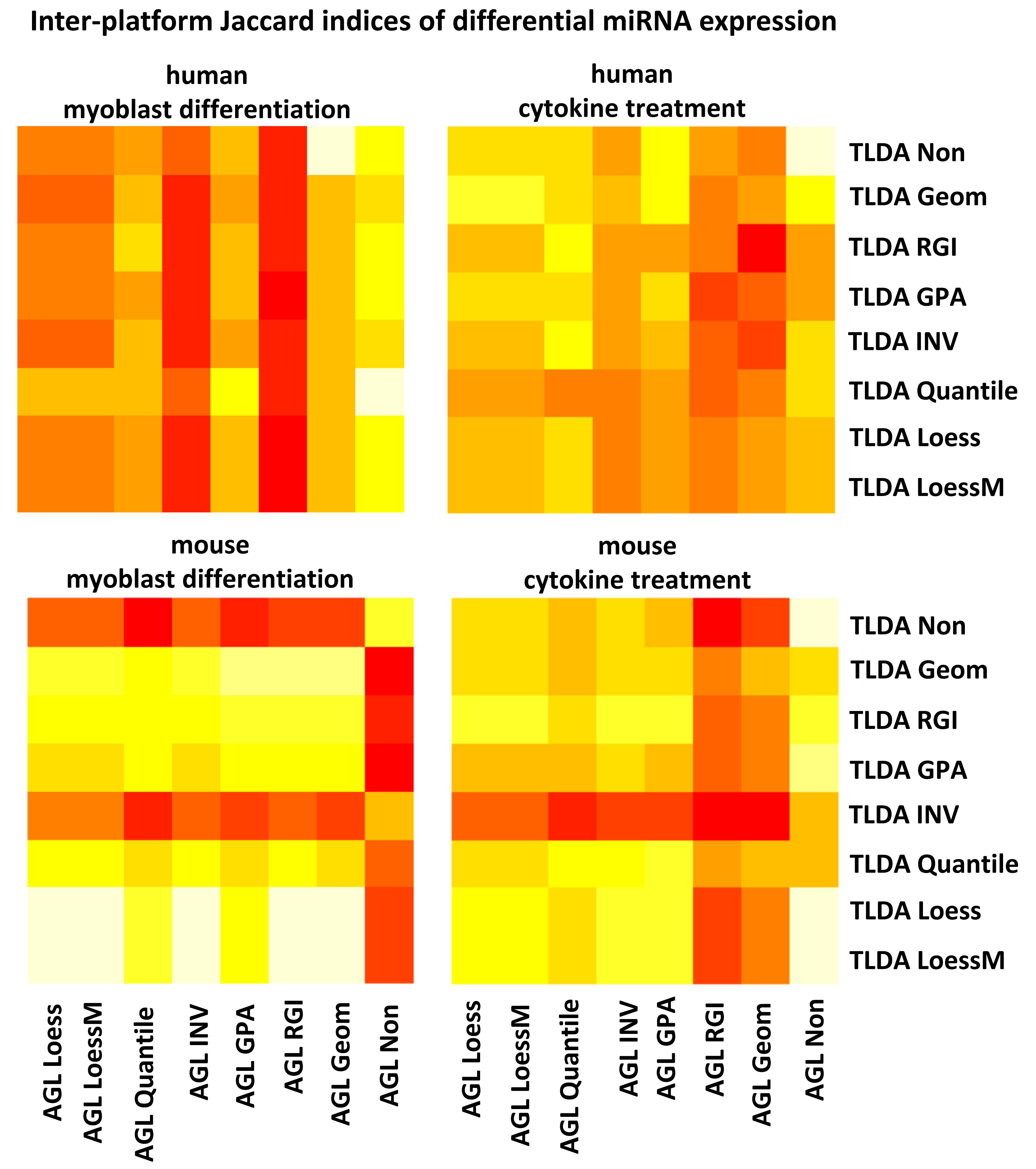

Supplement: Figure S4 — Heatmap of relative inter-platform similarity in detecting differential expression dependent on the normalization applied. Jaccard indices of significantly regulated miRNA overlap across the two distinctively normalized platforms, AGL array and TLDA card, were depicted as heatmap for myoblast differentiation and cytokine treated samples. Colour coding of the heatmap was as stated in Figure S2. (TIF) [file pone.0038946.s004.tif]

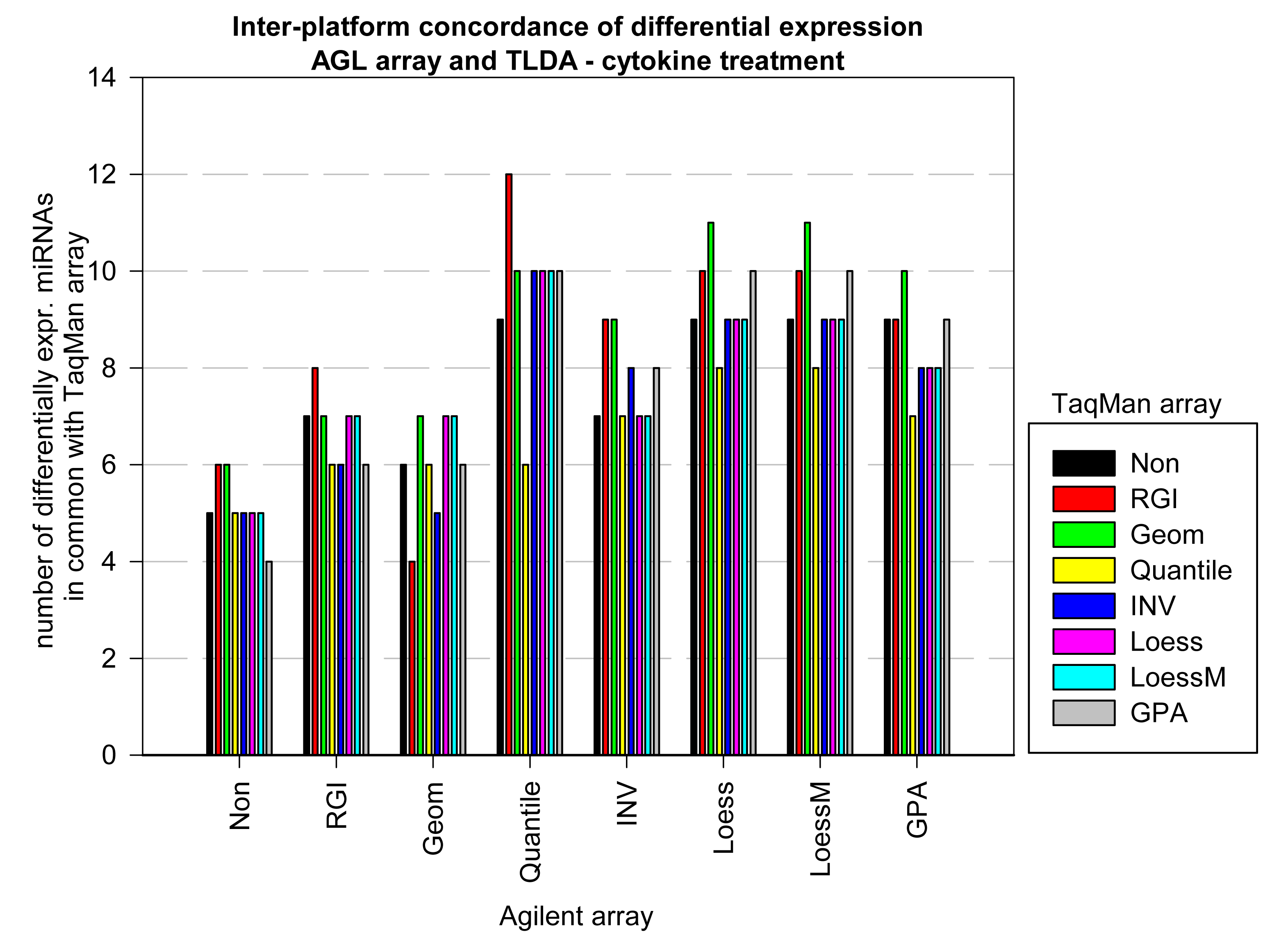

Supplement: Figure S5 — Inter-platform absolute concordance of differential expression upon cytokine treatment. Inter-platform concordance of differential expression detected by human AGL array and TLDA across different normalization methods and no normalization were exemplarily shown for the effect of cytokine treatment. The overlapping number of miRNAs between datasets was depicted for all possible inter-platform combinations of distinctively normalized datasets. (TIF) [file pone.0038946.s005.tif]

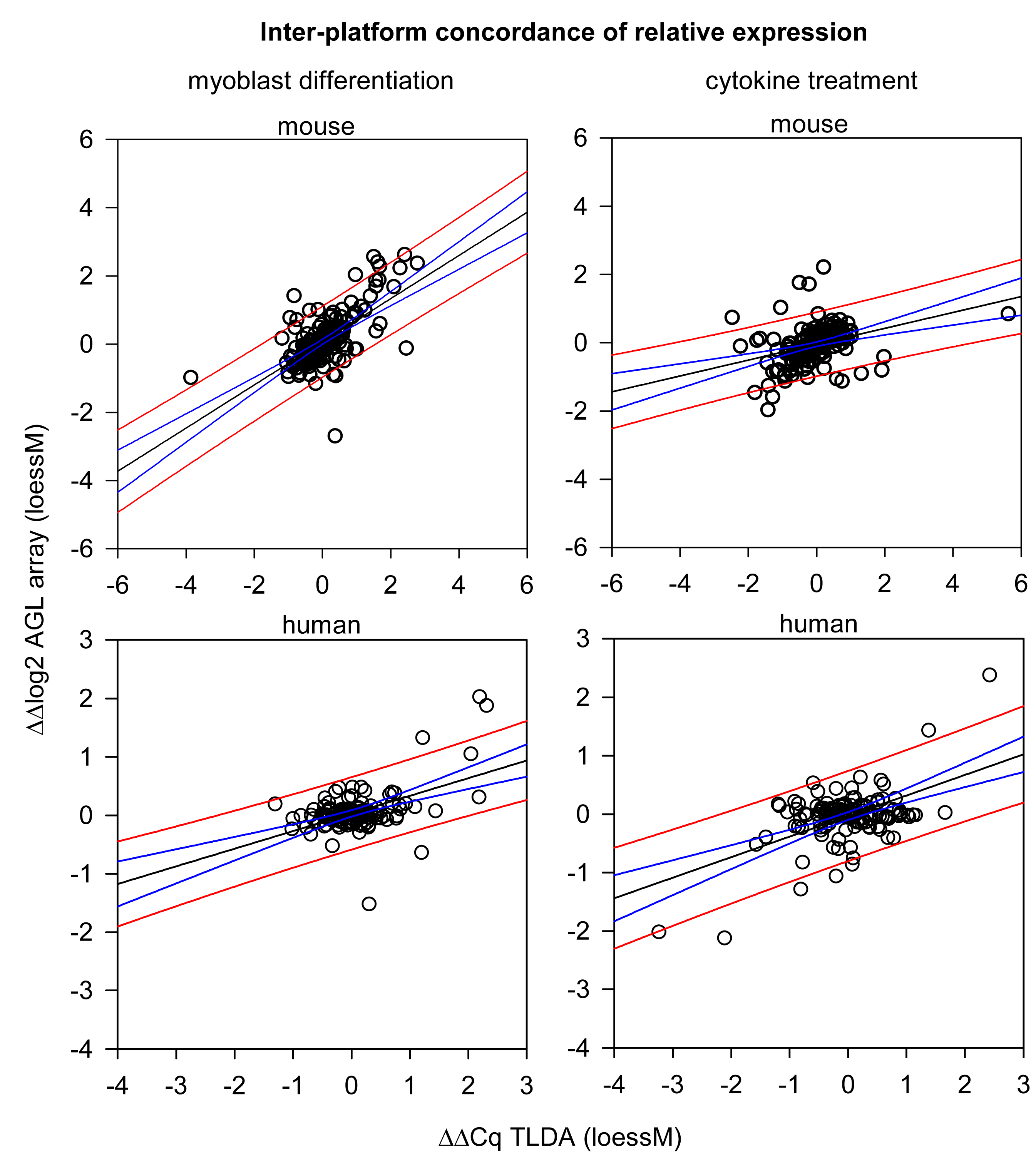

Supplement: Figure S6 — Fold-change compression by microarray profiling. Inter-platform fold-change concordance of human and mouse TLDA and AGL platform of the miRNA subset common on both platforms was illustrated by scatter plot of mean values of fold-changes (log2 scale or Cq, respectively). A fold-change compression of AGL platform values relative to the TLDA platform was indicated by linear regression (black line) shown with 95% confidence band (blue line) and 95% prediction band (red line). (TIF) [file pone.0038946.s006.tif]

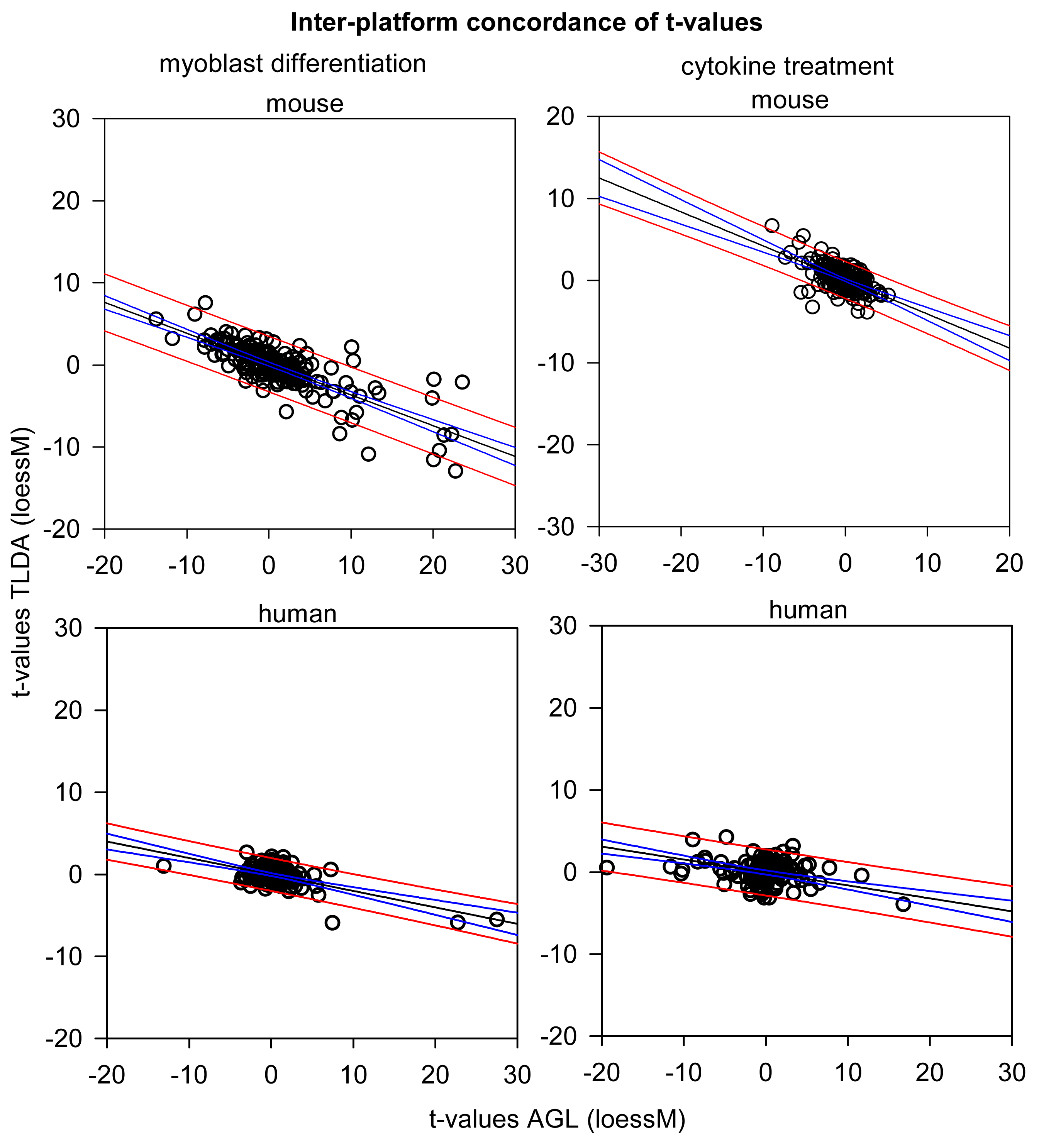

Supplement: Figure S7 — Inter-platform concordance of t-values reveals compression of t-values by qPCR profiling. Inter-platform concordance of t-values of human and mouse TLDA and AGL platform for the common miRNA subsets was illustrated by scatter plot. A compression of t-values of TLDA platform relative to the AGL platform was indicated by linear regression (black line) shown with 95% confidence band (blue line) and 95% prediction band (red line). (TIF) [file pone.0038946.s007.tif]
